# Macrophage-extracellular matrix interactions: Perspectives for tissue engineered heart valve remodeling

**DOI:** 10.3389/fcvm.2022.952178

**Published:** 2022-09-13

**Authors:** Nikolaos Poulis, Marcy Martin, Simon P. Hoerstrup, Maximilian Y. Emmert, Emanuela S. Fioretta

**Affiliations:** ^1^Institute for Regenerative Medicine, University of Zurich, Schlieren, Switzerland; ^2^Wyss Zurich, University and Swiss Federal Institute of Technology (ETH) Zurich, Zurich, Switzerland; ^3^Department of Cardiovascular Surgery, Charité Universitätsmedizin Berlin, Berlin, Germany; ^4^Department of Cardiothoracic and Vascular Surgery, German Heart Center Berlin, Berlin, Germany

**Keywords:** collagen, fibronectin, inflammation, regenerative medicine, scaffold functionalization, immune response, extracellular matrix (ECM), macrophages

## Abstract

*In situ* heart valve tissue engineering approaches have been proposed as promising strategies to overcome the limitations of current heart valve replacements. Tissue engineered heart valves (TEHVs) generated from *in vitro* grown tissue engineered matrices (TEMs) aim at mimicking the microenvironmental cues from the extracellular matrix (ECM) to favor integration and remodeling of the implant. A key role of the ECM is to provide mechanical support to and attract host cells into the construct. Additionally, each ECM component plays a critical role in regulating cell adhesion, growth, migration, and differentiation potential. Importantly, the immune response to the implanted TEHV is also modulated biophysically *via* macrophage-ECM protein interactions. Therefore, the aim of this review is to summarize what is currently known about the interactions and signaling networks occurring between ECM proteins and macrophages, and how these interactions may impact the long-term *in situ* remodeling outcomes of TEMs. First, we provide an overview of *in situ* tissue engineering approaches and their clinical relevance, followed by a discussion on the fundamentals of the remodeling cascades. We then focus on the role of circulation-derived and resident tissue macrophages, with particular emphasis on the ramifications that ECM proteins and peptides may have in regulating the host immune response. Finally, the relevance of these findings for heart valve tissue engineering applications is discussed.

## Introduction

Every tissue in the body has distinct extracellular matrix (ECM) composition, that arises from a unique combination of up to ∼300 different ECM (e.g., collagen subunits, proteoglycans and glycoproteins) and ECM-related protein components (e.g., secreted factors and ECM regulators) (1). The main role of the ECM is to provide structural support to each tissue type and organ. In addition, each ECM component also plays a direct role in controlling cell adhesion, regulating cell growth, migration, proliferation, and differentiation potential (2); the native heart valve ECM being no exception.

Due to the distinct three-layer ECM composition, the heart valve leaflet structure is able to withstand constant pressure changes during the cardiac cycle (3, 4). The ventricularis or atrial is side of the leaflets, those which are exposed to pulsatile shear stress, are composed of radially oriented elastin fibers and provide the elastic recoil needed for when the valve opens and closes (5). The middle layer, or spongiosa of the valve, is comprised of proteoglycans such as chondroitin sulfate, glycosaminoglycans, and sparsely packed collagen fibers, all of which mitigate compression forces when the valve is closed (6). The circumferentially aligned, densely packed, collagen-rich (mainly collagen 1 (COL1), but also COL3) fibrosa layer is situated at the outflow tract and is the main structure that provides strength and stiffness necessary for valve sufficiency (7).

Because the ECM is the core component of heart valve functionality, diseases that abrogate ECM function, such as genetic conditions (8–11) and calcific aortic valve disease (CAVD) (12, 13), can disrupt normal valve performance and often warrants valve replacement. CAVD is the most common valve disease and is a progressive degeneration ranging from non-obstructive valve thickening to severe valve calcification, which may result in impaired leaflet movement and eventually leading to valve stenosis (12, 13). CAVD development is an active inflammatory process that culminate with the release of matrix metalloproteinases (MMPs) and cathepsins that drive pathogenic ECM remodeling and calcium deposition (14, 15). This may result in severe valve insufficiency and/or stenosis that will ultimately necessitate replacement.

In most cases, treating these severe conditions requires a valve replacement procedure (16), either using mechanical or bioprosthetic valves, as extensively reviewed elsewhere (17, 18). Despite the remarkable heart valve prosthesis evolution (19), in particular with the advent of transcatheter techniques (20), several clinical and societal dilemmas remain (Box 1). Importantly, current clinical-grade heart valve replacement solutions lack the ability to remodel, repair and/or grow upon implantation, determining a high incidence of reoperation to replace the valve implant, in particular in the young cohort (<60 years old) (21).

**BOX 1 Remaining clinical and societal implications for heart valve therapies.**

• Heart valve replacement procedures of severely dysfunctional valves, either using mechanical or bioprosthetic valves, is expected to reach 850,000 implants annually by 2050 (22).

• Patients receiving a mechanical valve are subjected to life-long anticoagulant treatment to prevent thrombosis (23).

• For elderly patients, current guidelines recommend a bioprosthetic valve generated from glutaraldehyde-fixed xenogenic tissue (e.g., porcine valves or bovine pericardium). This prosthesis has an improved hemodynamic profile, reducing the need for anti-coagulation therapy (24).

• Bioprostheses have residual immunogenicity (i.e., xenogenic alpha-gal epitopes) that can cause chronic inflammation upon implantation (25, 26). This leads to degenerative failure, and limited durability of the prosthesis, causing the need for multiple re-interventions in the young (21, 24, 27).

• European health care costs for patients having a heart valve replacement exceed €1 billion annually (28).

*In situ* heart valve tissue engineering has been proposed as a promising solution to achieve “next-generation” heart valve prostheses with the potential for life-long durability (18). Remarkably, tissue engineered heart valves (TEHVs), manufactured using either decellularized allogenic/xenogeneic valves or bioresorbable polymer-based valves, have reached clinical translation (29–35). While wound healing and the foreign body response are amongst the most hypothesized mechanisms behind the integration and remodeling of TEHVs (36–38), less attention has been given to the role of ECM proteins in regulating remodeling upon implantation. Therefore, the aim of this review is to describe what is currently known about the interactions and signaling networks occurring between ECM proteins and macrophages, and how these interactions may impact the long-term *in situ* remodeling outcomes of tissue engineered ECM (TEM)-based implants. First, we provide an overview of *in situ* heart valve tissue engineering approaches with a particular focus on TEM-based TEHVs (section “*In situ* heart valve tissue engineering”). Then, we briefly summarize the fundamentals of the wound healing and remodeling cascades, specifically examining two of the first responders to injury, circulation-derived macrophages and resident tissue macrophages (RTMs) (section “The role of macrophages in tissue remodeling”). We then discuss the role of ECM proteins, such as collagens (COL), fibronectin (FN), and their corresponding peptides, in regulating the macrophage response, with particular emphasis on the implications for remodeling mechanisms (section “Immunoregulation of extracellular matrix proteins”). Finally, the relevance of these findings for heart valve tissue engineering applications is discussed by proposing examples of scaffold functionalization using ECM or ECM-related proteins to regulate the host immune response toward adaptive remodeling (section “Discussion: Relevance for *in situ* heart valve tissue engineering”).

## *In situ* heart valve tissue engineering

*In situ* tissue engineering relies on the regenerative potential of the recipient’s body to integrate and remodel an implanted off-the-shelf available construct. The scaffold used for this approach should withstand the native mechanical environment immediately upon implantation, favor host cell adhesion, migration and proliferation, support ECM production, and allow for adaptive remodeling toward a native-like functional living tissue (18, 36).

To achieve this, a multitude of different tissue engineering strategies have investigated distinct scaffold materials that ensure functionality and remodeling of the implanted TEHV, as extensively reviewed elsewhere (18). Briefly, TEHVs utilizing decellularized homografts have shown favorable long-term performance and limited *in situ* cell repopulation in clinical trials (30, 39–42). However, maladaptive remodeling phenomena (i.e., fibrosis and calcification) and not favorable long-term outcomes (i.e., 51% of freedom from reoperation at 10 years compared to 80% for standard cryopreserved allografts) have been reported (43–45).

TEHVs manufactured from decellularized xenografts have been associated with controversial results, with marked discrepancy between preclinical and clinical studies. Briefly, preclinical investigations in large animal models showed promising performance of xenograft-based TEHVs, with cellular infiltration throughout the tissue thickness (46). However, clinical trials were mostly unsuccessful, with signs of maladaptive remodeling [i.e., fibrosis, leaflet thickening, calcification, lack of cellularization, and chronic inflammation (47–53)], that may have caused the observed valve insufficiency and/or stenosis.

The functionality and remodeling potential of bioresorbable polymeric valves, which are compatible with surgical and transcatheter implantation techniques, were first demonstrated in large animal models (54–59). Within these studies, the valves showed acceptable functionality for up to 12 months, rapid cellularization, ECM deposition and progressive scaffold reabsorption (56, 58). Clinical translation of this approach is currently ongoing. A first pulmonary valve conduit design was evaluated in 12 pediatric patients [Xplore-1 study (34)], but moderate-to-severe pulmonary valve regurgitation was observed in 11 out of 12 patients (60). A retrospective analysis of the implanted conduits revealed leaflet thickness heterogenicity, with the leaflets being thinner in the commissural area. Therefore, the leaflet design was modified to achieve a more homogeneous thickness distribution. The improved of pulmonary valve conduit design has been then implanted in six children [Xplore-2 study (35)]. In this trial, moderate pulmonary valve regurgitation was reported for only 1 patient at the 12 months follow-up (60). In addition, 1 patient developed rapidly progressing stenosis and required conduit replacement (60). Given the mixed outcomes of these two clinical trials, as well as the reported intra-valve and inter-valve differences in remodeling and incomplete scaffold reabsorption after 1 year in preclinical studies (56, 58, 61–63), further data with longer follow-up time points are needed to determine the true clinical value of this approach. Alternatively, *in vitro* grown human cell-derived tissue engineered matrices (TEMs) have been recently proposed as a promising material to fulfill the need for an easily accessible and an off-the-shelf option for TEHV development (64, 65).

TEMs are obtained by *in vitro* culture of human fibroblast-like cells [e.g., human dermal fibroblasts or vascular-derived myofibroblasts (64–68)] on a biodegradable scaffold [i.e., fibrin gel or poly-4-hydroxybutirate (P4HB) coated polyglycolic acid (PGA) (64, 67–70)] for a pre-determined amount of time to have sufficient deposition of ECM proteins. After culture, the tissue is decellularized to ensure immunocompatibility and grant off-the-shelf availability, while also preserving the ECM structure (66). The resulting TEM can then be used to manufacture off-the-shelf available TEHVs to replace (pulmonary or aortic) heart valves with promising acute performance (64, 65), and sustained functionality, up to 1 year (67), with host cell repopulation, endothelialization, integration, and remodeling potential over time being consistently observed (67, 68, 70–74).

TEMs can be described as complex materials, comprised of a multitude of ECM proteins, minimal amounts of residual DNA that may remain from the decellularization process, and possibly bioresorbable polymer remnants (64, 65, 67, 68, 70, 71, 74). Upon implantation, each of these TEM components have a potential impact on the immune response to the implant and, thus, will contribute to its remodeling process (75); the ECM proteins being no exception. Generally, the ECM components of a TEM implant are considered for their mechanical properties (64, 71). Importantly, ECM proteins also have significant biocompatible properties that can guide cell behavior (76), and can potentially regulate macrophage polarization upon implantation.

## The role of macrophages in tissue remodeling

The remodeling of a tissue engineered construct is a highly complex and dynamic process characterized by the interaction of different immune cells with the implant, followed by the release of cytokines to further attract immune and specialized cells to the injury/implantation site (75). Little is known about the remodeling following an injury in native heart valve leaflets. Animal studies indicate surgically wounded valves remodel by recruiting activated macrophages, myofibroblasts, and endothelial cells that contribute to the deposition of proteoglycans and secretion of MMPs (77–79). Eventually, as a result of this healing cascade, a collagen-rich fibrous tissue is formed and, over time, the valve leaflet is re-endothelialized (79). Macrophages have been repeatedly reported as early responders that govern wound healing and remodeling processes by secreting growth factors, chemokines and cytokines [e.g., transforming growth factor beta (TGFβ), fibroblast growth factor (FGF), platelet derived growth factor (PDGF) and vascular endothelial growth factor (VEGF) (80)] as extensively reviewed elsewhere (81, 82). This occurs also in the heart valves, where by secreting TGFβ and FGF, macrophages play a key role in activating valvular interstitial cells (VICs), to favor proliferation and migration during valve repair and remodeling (83). However, aberrant TGFβ stimulation is also the first step to pathogenic fibrosis and/or osteogenesis, potentially leading to fibrotic valve stenosis and calcific valve disease (84). Therefore, there is a fine balance between the signaling molecules secreted by macrophages that are needed to decide the fate between fibrosis and regeneration of both native and TEHVs, making these cells one of the key players in the repair and remodeling cascades.

**FIGURE 1:**
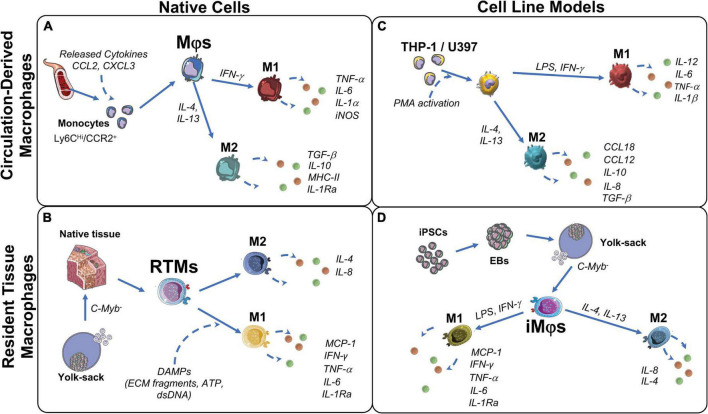
Origin and function of macrophages and their corresponding cell models. **(A)** Circulation-derived monocytes (CD14^Hi^/CCR2^+^), activated by C-C motif chemokine ligand 2 (CCL2) and C-X-C chemokine ligand 3 (CXCL3) from cells at the wound site, differentiate to macrophages (Mφs). When in contact with cell debris and ECM fragments, Mφs polarize to M1 to induce clearance of the damaged cells/proteins. M1- Mφs secrete pro-inflammatory factors to attract ECM producing stromal cells and restore tissue integrity. Over time, to resolve inflammation, Mφs polarize to M2. M2-Mφs secrete anti-inflammatory factors that favor remodeling and restore tissue integrity. **(B)** Resident tissue macrophages (RTMs) are derived from C-Myb^–^ hematopoiesis in the yolk-sac and reside in native fetal tissues, but persists into adulthood. Upon tissue damage, RTMs are activated by damage associated molecular patterns (DAMPs) and polarize to M1. M1-RTMs secret pro-inflammatory cytokines that recruit circulating monocytes and neutrophils, but then quickly polarize to the M2 state to promote tissue restoration and homeostasis. **(C)** Immortalized leukemic cells THP-1 and U937 are used to model peripheral blood-derived monocytes and Mφs. M1 polarization is induced by lipopolysaccharides (LPS), interferon (IFN)-γ with subsequent pro-inflammatory cytokine production interleukin (IL)-12, IL-6, IL-1β, and tissue necrosis factor (TNF)-α. M2 polarization is induced by IL-4 and IL-13 with subsequent production of IL-10, IL-8, CCL18, CCL12 and transforming growth factor (TGF)β. **(D)** iMφs are a proposed model of RTMs *in vitro*. Their C-Myb independent ontogeny is similar to RTMs and have shown to polarize in a similar manner to the M1 (LPS, IFN-γ) and M2 (IL-4, IL-13) states. EB, embryoid body; iNOS, inducible nitric oxide synthase; iPSCs, induced pluripotent stem cells; LPS, lipopolysaccharides; MCP-1, monocyte chemoattractant protein 1; MHC-II, major histocompatibility complex class II; PMA, protein kinase C activator.

Importantly, it is very likely that the host response to an implanted engineered valve will substantially differ from the innate remodeling following an injury, in particular if an implant leads to the chronic activation of immune cells and foreign body response. After 2–4 weeks from implantation, the surface properties of the implant will modulate the foreign body reaction, by affecting protein adsorption and early responding immune cells adhesion, thereby affecting the inflammatory cascade and the subsequent cellular recruitment and activation on the implant (85). Although it is currently difficult to predict the human immune response to implanted material, we present an overview of circulation-derived and resident tissue macrophages’ role in host immune response in the following sections. Traditionally, all macrophages were considered to be derived from the bone marrow through circulating monocytes (section “Circulation-derived macrophages”) (Figure 1A). More recently, it has been established that resident tissue macrophages (section “Resident tissue macrophages”) are derived from embryonic progenitors and can persist and proliferate throughout adulthood (86) (Figure 1B).

### Circulation-derived macrophages

Circulation-derived macrophages originate from monocytes (carrying markers CD14*^Hi^*, CCR2^+^ and CSFR2^+^) that circulate in a dormant steady state and are products of hematopoiesis (87, 88). Circulating monocytes are attracted to the surface of a wounded area and/or implant, where they differentiate to macrophages (87, 88) (Figure 1A). These macrophages remove cell debris as well as clear foreign materials (82). In addition, they enable collagen deposition in the injured area, or on the implanted biomaterial, in order to induce tissue restoration and support further immune cell and fibroblast recruitment *via* cytokine release (88, 89).

The cytokines secreted in the early stages of wound healing are highly dependent on the biochemical cues present in the wound or on the surface of the implanted material. Several determinants, such as the presence of cell debris, external pathogens, ECM proteins, and foreign materials (i.e., bioresorbable polymers), can impact whether the circulation-derived macrophages display a pro- or anti-inflammatory phenotype (90). Pro-inflammatory polarization, primarily comprised of M1 sub-populations, enables the formation of multinucleated foreign body giant cells (FBGCs) with the intent to engulf and clear any foreign material and remove cellular debris. Normally, when M1 macrophages have concluded the clearance of cell debris and foreign bodies, further M1 polarization is not required and the cells repolarize into an anti-inflammatory state, generally known as the M2 state. Anti-inflammatory polarization is comprised primarily of M2 (e.g., M2a, M2b, M2c, and M2d) macrophage subgroups, and promotes tissue regeneration by secreting anti-inflammatory cytokines such as interleukin 10 (IL-10), IL-4, IL-1Ra and growth factors like TGFβ1, thereby attracting fibroblasts to restore tissue integrity (91, 92). Both M1 and M2 are considered dynamic states and with inherent plasticity that depends on the stimulation they receive from their surrounding environment or the presence of paracrine factors (92), thereby enabling the spontaneous initiation and resolution of inflammation. Importantly, macrophages guide *de novo* ECM synthesis by recruiting ECM producing α-smooth muscle actin (αSMA)-positive cells, but also by directly depositing ECM proteins (89, 93). ECM production by macrophages is primarily influenced by polarization toward the M2 subgroups (92). *In vitro* studies have showcased the direct production of collagens (COLVI and COLVIII) from stimulated monocytes and macrophages (94), as well as glycosaminoglycans and α-elastin in a strain-dependent manner, identifying macrophages as direct regulator of ECM turnover and synthesis (95). *In vivo* studies focusing on the cardiac wound healing response demonstrated that macrophages can directly deposit collagen in scar formation upon cardiac injury (96), further highlighting the role of macrophage-ECM crosstalk that should to be taken into consideration for TEHV implantation. M2 macrophages also enable ECM production and turnover at the wound/implantation site indirectly. For example, IL-10 and TGFβ1 secretion recruits and activates fibroblasts leading to ECM deposition in a myocardial infarction model (97). Furthermore, the paracrine effects of TGFβ1, PDGF, and MMP secretion have been shown to activate ECM producing cells, such as fibroblasts, to favor tissue repair and remodeling (96). However, the regulation of direct collagen synthesis from M2 macrophages remains unclear.

Thus, adaptive or maladaptive remodeling of a tissue engineered implant is a complex process highly dependent on the inflammatory response and, more specifically, on macrophage polarization. In the case of TEM-based cardiovascular implants, a multitude of ECM proteins such collagens, glycoproteins and proteoglycans are in direct contact with the circulation-derived macrophages through a network of signaling proteins such as integrins (85). These interactions may impact the long-term remodeling outcomes of *in situ* regeneration, and will be further detailed in section “Immunoregulation of extracellular matrix proteins.”

### Resident tissue macrophages

While circulation-derived macrophages originate from blood monocytes, resident tissue macrophages (RTMs) can originate from: (a) embryonic development, independent of hematopoiesis, and from (b) infiltrating monocytes that arise from bone marrow hematopoiesis in adulthood (98). Compared to circulation-derived macrophages, for RTMs from embryonic origin, primitive and transient hematopoiesis is c-Myb independent (99) (Figure 1B). In adulthood, RTMs reside in all tissues and are responsible for tissue homeostasis and immune surveillance with distinct functions specific for each tissue’s microenvironment (100). However, the exact origin of adult RTMs and the mechanisms of RTMs maintenance within the adult tissue are currently not clearly defined (101).

RTMs possess self-renewal capacity and, upon tissue injury, are rapidly activated by signals released from damaged cells (i.e., calcium, ATP, H_2_O_2_, and DNA) (100). As the first responders in the wound healing cascade due to their proximity to injured tissue, activation of RTMs enables immediate pro-inflammatory cytokine release (i.e., tissue necrosis factor (TNF)-α, inducible nitric oxide synthase (iNOS), and IL-1β), which then recruits neutrophils and monocytes (102). As soon as RTMs initiate the pro-inflammatory response, they start producing ECM in order to recover tissue integrity and provide supportive matrix for cell infiltration (102). Due to their constant interaction with ECM, RTMs play an integral role in tissue remodeling for *in situ* regeneration (88). In addition to circulation-derived macrophages, RTMs may also play a role in remodeling implanted materials. When a foreign material, like the hTEM, is implanted, RTMs may be capable of infiltrating into the implant from adjacent tissues, including the heart muscle and vascular wall. Whether RTMs promote or resolve rapid inflammation and signal adaptive tissue remodeling upon contact with TEM-based implants, still remains to be further explored in the future. In terms of TEHVs, cells are known to infiltrate the implant from the arterial wall (38). However, whether RTMs may undergo such migration has not been elucidated to this date.

The role of macrophages in cardiovascular tissue development, homeostasis, and disease, has been recently examined mostly murine models, as reviewed elsewhere (103). In regards to heart tissue, cardiac-resident macrophages play a key role in homeostasis as well as in limiting damage extension following a cardiac injury, resulting in necrotic and apoptotic cell clearance, promotion of angiogenesis and reduction of inflammation (98). In the heart valve, CD45^+^ cells identified in the leaflet were originally hypothesized to differentiate toward VICs (104). However, further analyses have identified an immune population with macrophage characteristics in these CD45^+^ cells (105, 106). In addition, adult and developing heart valves have revealed the presence of multiple immune cell populations with characteristic markers of RTMs (106–108). Similar to cardiac RTMs, valvular RTMs have a role in tissue homeostasis, and have been associated to ECM remodeling, aging, and disease (106–108). Harnessing the characterization of these cell types in native heart valves, as well as in TEHVs, would greatly improve our understanding of *in situ* regeneration and remodeling.

Although RTMs have been thoroughly investigated and characterized through lineage tracing experiments in mouse models, their detailed function has not been elucidated for every organ system in humans (109). In humans, the functional characterization of these cells has been limited primarily because RTMs are challenging to isolate, due to the minimal number available in the tissues (109). The use of macrophage cell models is therefore currently being proposed as a solution to combat these challenges (section “*In vitro* macrophage models”).

### *In vitro* macrophage models

Macrophage behavior *in vitro* has been investigated through a variety of cell lines isolated from human donors. Peripheral blood mononuclear cells (PBMCs) are the most accessible source of macrophages and have been studied extensively to assess the early steps of the remodeling cascade, early ECM production as well as material immunocompatibility in the cardiovascular tissue engineering field (95, 110–115); their advantages and disadvantages have been extensively reviewed elsewhere (116). However, human monocytes and monocyte-derived macrophages lack *in vitro* proliferation potential, leading to the constant need for new donor material (87). This disadvantage coupled with the inter-donor variability prompted researchers to use immortalized cell lines from a similar origin, such as leukemic immortalized monocytes THP-1 and U937 cells (87, 117) (Figure 1C). Compared to PBMCs, the high proliferation rate of these cell lines make them a practical source to study monocyte and macrophage function and differentiation, manifested by their broad *in vitro* applications for immune modulation approaches and in the cardiovascular tissue engineering field (117–119). However, there have been highlighted differences between THP-1 and PBMCs. Some suggest to limit the use of THP-1 cells when studying polarization, as THP-1 cells have a bias toward phagocytosis and the M1 phenotype (116, 120). Moreover, the malignant genetic background of these immortalized cells puts their application relevance into question. Especially as *in vivo* studies would still be required to validate any *in vitro* experiments using immortalized cell lines.

Due to RTMs paucity in tissues and limited availability of human donor materials, isolation in large numbers has been an unconquered milestone. Recently, iPSC-derived macrophages (iMφs) produce c-Myb independent macrophage-like cells from yolk sac-like structures (Figure 1D) and have been proposed to accurately mimic RTM biology *in vitro* (121–124). So far, due to their plasticity and adaptation potential to environmental stimuli, tissue specific resident-like iMφs such as alveolar-like, brain tissue resident-like (microglia-like), skin resident Langerhans-like, and liver resident Kuppfer-like iMφs have been successfully produced (123, 125, 126). However, research is still ongoing into iMφs that are functionally similar to cardiovascular RTMs. The use of iMφs may prove to be valuable for *in vitro* modeling of RTMs in wound healing and remodeling processes, specifically for *in situ* regeneration approaches.

## Immunoregulation of extracellular matrix proteins

The multi-step process of remodeling is not only controlled biochemically by cytokines, but also biophysically by cell-ECM interactions (76, 127). Hence, implants manufactured from decellularized ECM, such as TEMs, may actively influence immune cell (i.e., macrophages) behavior *via* integrin- or non-integrin mediated signaling.

Integrin-mediated cell-ECM interactions occur when transmembrane integrin receptors, such as focal adhesions, physically bind to different ECM components and lead to signal transduction mechanisms (2, 128, 129). These connections allow cells to perceive changes in the ECM microenvironment (e.g., composition, stiffness, and orientation), as well as to migrate through the ECM (130). TEM-based TEHVs have been reported to contain ECM proteins like collagens (COLI and COLIII), FN, as well as glycosaminoglycans (64, 65), therefore having a potential role in regulating macrophage response (64, 65), which should be further investigated.

Non-integrin mediated cell-ECM interactions are observed in the presence of bioactive ECM peptides or epitopes, also known as matrikines or matricryptins, generated by ECM fragmentation (2, 131). Both matrikines and matricryptins refer to ECM peptides that are usually not exposed in intact and mature ECM fibers, but that are able to regulate cellular activity when exposed after degeneration. These ECM sites become available only after structural or conformational alterations (i.e., enzymatic degradation, self-assembly, denaturation, cell-mediated mechanical forces, and adsorption to surfaces) to the ECM proteins (132). In the field of tissue repair and regeneration, it is possible to identify these ECM transformations at the site of tissue injury, suggesting that the consequent exposure of specific ECM peptides may provide new signaling cues to regulate immune cells and tissue restoration (132). It is to be expected that these protein and relative fragments are also similarly present at the site of implantation of a tissue engineered implant, as well as they may be contained in a TEM-based valve replacement. Importantly, these molecules form a class of damage-associated molecular patterns (DAMPs) that activate immune (e.g., macrophages) and differentiated (e.g., fibroblasts) cells *via* toll-like receptor (TLR2 and TLR4) signaling (133). This alerts the immune system to tissue damage and initiates tissue repair (2, 76, 134, 135). Among the ECM peptides discovered to be capable of acting as DAMPs, there are those derived by FN, fibrinogen, versican, and heparan sulfate. Overall, these peptides engage with multiple pattern recognition receptors and initiate a pro-inflammatory response (136). As an example, biglycan and decorin have been reported to be capable of both generating a pro-inflammatory response in macrophages by interacting with TLR2 and TLR4, as well as promoting an anti-inflammatory response critical for the resolution of inflammation (137–139). In addition, bioactive ECM fragments from the basement membrane have been identified in both healthy and diseased hearts, and associated to cardiac disease (140). An example is endostatin, one of the most investigated peptides derived from enzymatical cleavage of COLXVIII. Endostatin is mostly known for its anti-angiogenic properties, but also anti-fibrotic and anti-tumor effects, *via* the reduction of anti-inflammatory cytokine (e.g., IL-4, IL-10, IL-13, and VEGF) expression and of M2 polarization (141).

**FIGURE 2:**
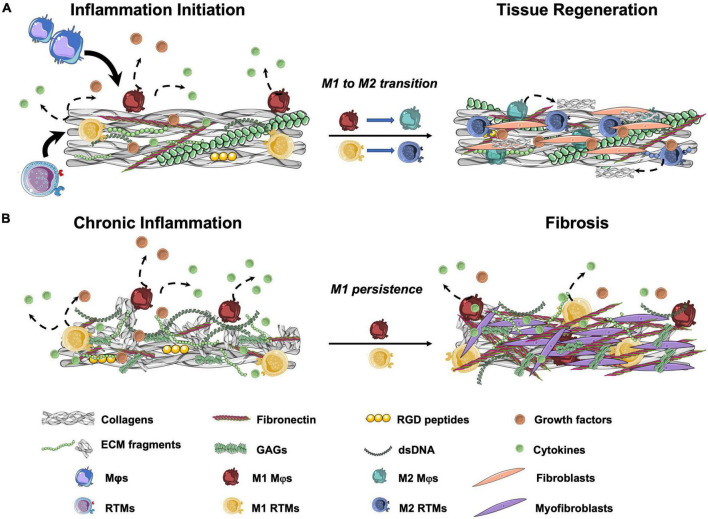
Schematic representation of wound healing outcomes after tissue injury. **(A)** In normal wound healing cascades, resident tissue macrophages (RTMs) and circulation-derived macrophages (Mφs) are attracted to the damaged area. When in contact with cell debris and ECM fragments, RTMs and Mφs polarize to the M1 state and produce pro-inflammatory cytokines. After the initial pro-inflammatory response, M2 polarization with progressive resolution of the inflammation occurs. Among other stromal cells, fibroblasts are recruited to site to restore tissue integrity. **(B)** In the presence of a high amounts of ECM fragments, peptides, cell debris and/or DNA remnants, prolonged M1 polarization with limited or absent M2 transition occurs. This results in a chronic pro-inflammatory stimulation that leads to the production of unorganized collagen-rich ECM by the activated fibroblasts (myofibroblasts) and may cause tissue fibrosis. GAGs, glycosaminoglycans; RGD, Arg-Gly-Asp.

In the following sections, we will discuss what is known about the effect that ECM proteins (such as collagen and FN, that have been found in TEM-based TEHVs) and ECM-derived peptides have on macrophages and their relevance for repair and remodeling mechanisms (Figure 2).

### Macrophage/collagen interactions

Collagens are the most abundant proteins in the human body (142) and one of the major components of *in vitro* grown TEMs. Collagens provide structural and mechanical properties to the tissue, while also playing an important role in controlling and regulating immune cells (143). In this regard, macrophages have been shown to be key players in controlling collagen homeostasis by contributing to collagen degradation and turnover (144), but also collagen synthesis *in vitro* and *in vivo* (e.g., tissue fibrosis during heart repair) (96, 144).

The immunomodulatory effect of collagen is a consequence of the different ligands that can be recognized by immune cells [e.g., integrins, discoidin domain receptors, immunoglobulin-like receptors, and mannose receptors (88, 96)]. These interactions have been reported to increase cell adhesion and integrin expression in both innate and adaptive immune cells (145), and reduce the inflammatory response of macrophages *via* the leukocyte-associated immunoglobulin-like receptor 1 (LAIR-1 or CD305) (146, 147). In addition, macrophage migration and mechanosensing in a fibrillar collagenous matrix is controlled by α2β1 integrin binding and stretch-activated channels, resulting in macrophage migration toward the source of a dynamic force, such as the substrate deformation caused by contractile fibroblasts in the tissue (146, 148, 149). Finally, *in vitro* studies showed that macrophage infiltration into collagen-based substrates is impacted by collagen architecture (150). Taken together, these findings suggest that collagen structure and organization in tissue engineered products, such as TEM-based TEHVs, may influence adhesion, infiltration and migration of macrophages, and potentially mediating the host’s inflammatory response.

### Macrophage/fibronectin interactions

FN is a large glycoprotein that can be either present in a soluble [e.g., plasma FN (129)] or non-soluble form (e.g., cellular or tissue FN) that is produced by cells such as fibroblasts and endothelial cells (151). FN fibrils are required for the deposition of collagen and for the binding of glycosaminoglycans, thereby becoming a fundamental protein to ensure ECM remodeling during the wound healing cascade (152). FN, also one of the main ECM components of TEM-based implants, has been reported to influence the adhesion, migration, and apoptosis of monocytes and fibroblasts, among other cells (151, 153). Monocyte differentiation into macrophages was enhanced when FN-coated surfaces were used, suggesting an important role for integrin-mediated adhesion in macrophage differentiation (154, 155). FN has also demonstrated influence of macrophage polarization toward a pro-inflammatory state, characterized by increased phagocytosis activity (155, 156). In addition, the pro-inflammatory effect was further elicited in macrophages that interacted with both collagen and FN, a situation that simulates cases of tissue damage where numerous ECM proteins are exposed or fragmented (157).

Based on these results, it is expected that TEM-based TEHVs, derived by *in vitro* culture of fibroblast-like cells, have high non-soluble FN content that may therefore influence macrophage polarization toward a pro-inflammatory status. This will lead to the recruitment and activation of more immune cells and ECM-producing αSMA^+^ cells, similar to what is commonly observed in a wound healing response (81, 82). In theory, macrophages will then play a key role in attracting fibroblast-like cells to stimulate TEM-based TEHV remodeling (83).

### Macrophage/extracellular matrix-derived peptide interactions

ECM-derived peptides are bioactive sites of ECM proteins such as collagens, FN, and elastin, also referred to as matrikines and matricryptins. Peptides may either be generated by direct ECM damage (e.g., upon injury), or be exposed in immature ECM proteins (e.g., in a remodeling tissue) (2, 131). Importantly, these peptides are considered DAMP molecules that may have the potential to activate immune cells and/or stromal cells (e.g., fibroblasts) to initiate tissue repair through influencing cell migration, adhesion, and differentiation both *in vitro* and *in vivo* (2, 76, 134, 135).

RGD (Arg-Gly-Asp) is an integrin-binding peptide that results from exposed collagen, FN, vitronectin, and osteopontin proteins upon conformational changes as its active domain becomes apparent only upon substrate denaturation of collagens (158, 159), or absorption of FN and vitronectin (160). In areas of tissue injury, FN fibrils were shown to have an increased affinity for the exposed RGD sites of denatured collagens. It is hypothesized that this specific ECM composition, with abundance of exposed RGD sites as both proteins carry this sequence, is recognized by the immune system as a unique wound signal (161). Hence, it is not surprising that macrophages are affected by the presence of RGD domains, which stimulate the adhesion and M2 polarization both *in vitro* and *in vivo* (162). On the other hand, macrophage adhesion and fusion to FBGCs is supported by the presence of RGD peptides in combination with another FN-derived peptide, PHSRN (Pro-His-Ser-Arg-Asn) (163), which suggests that a combination of matricryptins—that could be found in a site of tissue injury—may be important in regulating a pro-inflammatory response. Finally, the inflammatory profile of macrophages and consequent fibrotic tissue formation could be mitigated when the RGD-binding integrins were blocked using specific antibodies (147), once again suggesting a pro-inflammatory effect of this peptide. Taken together, these results indicate a very active but controversial role of the RGD peptide in modulating macrophage response and polarization, making this peptide an interesting target for biomaterial functionalization (149).

VGVAPG (Val-Gly-Val-Ala-Pro-Gly) is an elastin-derived peptide that becomes exposed upon elastase and MMP12 digestion of elastin fibers. This peptide has repeatedly been shown to have chemotactic properties for monocytes, macrophages, and fibroblasts (164–166), and is also involved in ECM degradation *via* regulation of MMP expression (167). Specifically, the presence of elastin-derived fragments is associated with increased proliferation and decreased elastin synthesis in vascular smooth muscle cells (168). In addition, the VGVAPG peptide increased smooth muscle cell migration through the elastic lamina, thereby leading to intimal hyperplasia (168, 169). Elastin is one of the key components of vascular, valvular and heart tissue, highlighting the importance of these results for clinical translation in the cardiovascular field.

PGP (Pro-Gly-Pro) is a matricryptin derived from COL1 that plays an important role in mediating inflammation. This peptide has sequence and structural homology with one of the domains on alpha chemokines and, therefore, can mimic their chemotactic effect in inflammation models (160, 170, 171). Some bioactive ECM peptides are also involved in ECM synthesis and remodeling. Among these, the peptide GHK (Gly-His-Lys) was reported to favor wound healing and skin regeneration when combined with copper ions (Cu^2+^), by stimulating collagen turnover, modulating MMP activity, and attracting immune cells to the wound (172).

Taken together, these studies highlight the importance of ECM and ECM-derived peptides in regulating the early steps of the inflammatory response by directly affecting macrophages (summarized in Figure 2). Because collagens and FN are among the main components of the TEM, the presence of such peptides should be carefully evaluated to better understand the intricated steps of tissue remodeling upon implantation. The possibility of using ECM proteins and fragments to favor the TEHVs *in situ* remodeling potential may be an interesting strategy to influence the remodeling cascade as well as favor tissue integration and adaptive remodeling of the implant, as further discussed in section “Discussion: Relevance for *in situ* heart valve tissue engineering.”

## Discussion: Relevance for *in situ* heart valve tissue engineering

The importance of ECM proteins in regulating macrophage behavior and, therefore, the consequent remodeling cascade should be considered when developing a TEHV. Macrophages are the primary mediators of host engraftment and will drive the response to the different biomaterials implanted (142), potentially deciding the fate of TEHV remodeling. Based on this, researchers are continuing to investigate ways to improve the remodeling of TEHVs with *in situ* regenerative potential.

### Improving the characterization of decellularized tissue engineered matrices-based tissue engineered heart valves

Originally developed to achieve an off-the-shelf available immunocompatible product and to limit leaflet retraction observed in autologous cell-based TEHVs (173–175), decellularized TEM-based TEHVs have been developed. However, the TEM-based TEHVs have been reported to undergo adverse remodeling in chronic studies implanted in sheep, with leaflet thickening and shortening caused by fusion of the leaflet to the wall, and resulting in severe valvular insufficiency within 24 weeks after implantation (68, 73, 74). A possible explanation for this outcome was obtained by using computational modeling to simulate stress and strain distribution on the valve leaflet. Sanders et al. showed that the simplified geometry of TEM-based TEHVs led to radial leaflet compression when subjected to physiological (pulmonary) pressure conditions (176). Based on this, further computational simulation was used to identify an improved valve geometry that could counteract the observed leaflet retraction (176). To impose this geometry, TEM-based TEHVs were manufactured using a constraining bioreactor insert during tissue culture and tested in a preclinical sheep model (67). Upon implantation, the TEHVs demonstrated *in vivo* performance for up to 1 year, and underwent native-like remodeling. Remarkably, no signs of adverse remodeling (i.e., leaflet thickening or leaflet-wall fusion phenomena) were observed (67). The morphological evaluation was indicative of functional native-like remodeling, with thin and shiny leaflets, complete integration within the adjacent native pulmonary artery wall, and formation of a neo-sinus. On a microscopic scale, the authors reported extensive cellular repopulation of the entire valve, improved matrix composition with elastin deposition, as well as reorganization of the collagen fibers (67). Taken together, these results indicate that valve geometry is one of the key factors in ensuring long-term TEHV function. In a subsequent study, Motta et al. investigated how the computational-inspired TEHV geometry could impact the host cell response and, therefore, tissue remodeling (177). Focused on macrophages, αSMA-positive cells, and endothelial cells as key players of the remodeling cascade, the authors found that compared to the first simple, non-physiological geometry (74), the remodeling of computational modeling-inspired TEHVs having a physiological-like design had negligible amounts of macrophages and αSMA-positive cell infiltration, had the absence of thickening, and showed rapid endothelialization (177).

**FIGURE 3:**
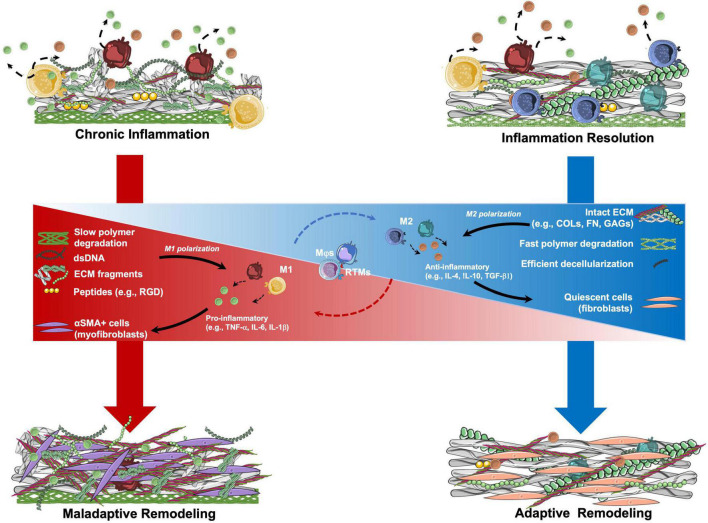
Hypothetical mechanisms involved in the remodeling of tissue engineered matrix (TEM)-based implants. TEM composition may be a crucial parameter in determining the remodeling potential of TEM-based implants. Resident tissue macrophages (RTMs) and circulation-derived macrophages (Mφs) are key mediators of the inflammatory process. Once in contact with TEMs, RTMs and Mφs may start a pro- or anti-inflammatory signaling by polarizing between M1 and M2 states, respectively. The sustained presence of polymer remnants, ECM fragments, and double stranded DNA (dsDNA) residues from incomplete decellularization, may lead to pro-inflammatory stimulation of RTMs and Mφs with consequent M1 polarization with the release of pro-inflammatory factors. This pro-inflammatory state may lead to the activation of fibroblasts into myofibroblasts, with the consequent production of a collagen-rich ECM, similarly to what is observed in tissue fibrosis (maladaptive remodeling). In contrast, TEMs having intact ECM proteins (e.g., COLs, FN, GAGs) as well as low amounts of dsDNA after optimal decellularization, may determine a short pro-inflammatory phase followed by M1 polarization resolution and transition to the M2 state. M2 RTMs and Mφs may help with provisional matrix formation. Recruited fibroblasts populate the implant and produce organized ECM until resolution of the inflammation occurs (adaptive remodeling). αSMA, alpha smooth muscle actin; COLs, collagens; FN, fibronectin; GAGs, glycosaminoglycans; IL, interleukin; RGD, Arg-Gly-Asp; TGF, transforming growth factor; TNF, tissue necrosis factor.

However, we can hypothesize that, other than TEHV geometry, also TEM composition may influence macrophage response and, therefore, the remodeling cascade, as summarized in Figure 3. We have previously described how ECM composition and integrity can play a role in regulating macrophage response (section “Immunoregulation of extracellular matrix proteins”). In addition, the presence of scaffold remnants may induce a foreign body response, with macrophage activation and possibly fusion to form FBGCs, as reviewed elsewhere (178). Finally, residual cellular components (i.e., DNA), may influence macrophage response and, therefore, the remodeling cascade, as detailed below.

The immunocompatibility of biomaterials made from decellularized (native or *in vitro* grown) tissues may be affected by the degree of decellularization, as the presence of cell debris may influence the immune response upon implantation. Decellularization protocols, as reviewed elsewhere (179, 180), generally aim at lysing resident cells to drastically reduce the immunogenic components of the starting tissue (i.e., DNA remnants) using a combination of chemical, physical, and enzymatic reactions, all while preserving the ECM. However, complete removal of all cellular components has not been shown so far. The presence of several antigens and/or DAMPs released from lysed cells (e.g., calcium-binding proteins, DNA, ATP, chromatin, nuclear proteins), which has been reported to potentially cause an adverse immune response upon implantation (181). Therefore, decellularized tissues should comply with the quantitative criteria described in 2011 (182): no visible cell nuclei in H&E or DAPI staining; double stranded DNA content < 50 ng/mg of dry tissue; and DNA remnant size < 300 base pairs. It has been shown that residual cellular debris, such as DNA, mitochondria and cell membrane proteins, can promote a pro-inflammatory M1 macrophage phenotype both *in vitro* and *in vivo* (183). This is particularly striking as clinical-grade biological decellularized tissues have great variations in the amount of retained cell remnants, a parameter that may cause differences in the tissue remodeling outcome upon implantation and device efficacy (184). Indeed, the DNA amount and degree of fragmentation within decellularized tissues was reported to influence macrophage phenotype both *in vitro* and *in vivo*, with a more effective decellularization being associated with a shift from an M1 to M2-like phenotype (185, 186).

On the other hand, extensive decellularization protocols can alter the three-dimensional structure of the ECM proteins [i.e., glycoproteins, proteoglycans, fibrinogen and fibronectin domains, tenascin c, etc. (136)], thereby creating further DAMPs (187). Therefore, maintaining the integrity of ECM proteins upon decellularization is important to ensure not only sufficient mechanical properties of the tissue, but also to promote an anti-inflammatory effect (185).

Several *in vitro* studies have investigated how decellularized native tissues can modulate macrophages polarization. For example, ECM scaffolds obtained from decellularized porcine small intestine submucosa (SIS) proved to favor a M2-like macrophage phenotype, with anti-inflammatory and pro-remodeling characteristics (183, 186, 188). On the other hand, dermal tissue-derived ECM scaffolds promoted a pro-inflammatory M1-like phenotype (183). However, the cause of macrophage polarization variation on the different substrates was not clarified. Importantly, macrophage polarization is often not quantitatively apparent *in vivo* and results may differ significantly between pre-clinical and clinical data (186). In this regard, the clinical use of decellularized porcine, SIS-based or valve-based, TEHVs resulted in severe adverse events for pediatric applications (47, 53). Remarkably, such an adverse effect was not observed during pre-clinical investigations in sheep models (189–192), as extensively reviewed elsewhere (18). These results suggest that decellularized xenograft-based valvular replacements may still contain α-galactose (25), a pro-inflammatory epitope that, particularly in pediatric patients, may have caused the adverse inflammatory response resulting in valve calcification and degeneration (47, 53). Therefore, independent of tissue origin, xenograft material may elicit a strong inflammatory response with detrimental consequences.

Taken together, these results highlight the limitations of both *in vitro* and *in vivo* preclinical models and suggest the need for an improved testing platform to assess immunocompatibility of decellularized products.

### Extracellular matrix-based functionalization of bioresorbable polymeric tissue engineered heart valves

Among the different types of TEHVs with *in situ* remodeling potential, polymer-based TEHVs have generated considerable interest and their clinical translation is currently ongoing with however, mixed outcomes so far, as discussed in section “*In situ* heart valve tissue engineering.” While conceptually, such materials may have multiple advantages (e.g., reasonable cost, scalability, tenability of degradation, mechanical properties and architecture), long-term safety and efficacy of this concept, especially when the initial polymer is fully resorbed, remains to be elucidated.

Nevertheless, researchers are constantly trying to improve their polymer-based scaffolds by promoting cell recruitment and endogenous tissue formation. One way to achieve this goal is to implement microstructural design features (i.e., pore size or polymer fiber orientation) to resemble native ECM architecture of the heart valve (62, 193), as reviewed elsewhere (38). Scaffold microarchitecture can be further combined with polymer functionalization using ECM proteins and ECM-derived molecules (55, 194). It was found that non-covalent scaffold functionalization was successfully achieved by developing a hybrid polymer, which combined the durability of synthetic polymers with the biocompatibility of gelatin (55). By using a cell-free rapid jet-spinning manufacturing process, the resulting TEHV had not only mimicked the fibrous, anisotropic architecture of the native leaflet with the synthetic polymers, but also had enhanced implant biocompatibility that favored cellular attachment and infiltration with the use of gelatin. These gelatin-functionalized TEHVs have been successfully implanted in an acute sheep model, demonstrating functionality *in vitro* as well as *in vivo* (55). However, long-term comparative studies are needed to understand the benefits of gelatin functionalization may have on the remodeling potential of this hybrid TEHV.

Considering its significant role in mediating macrophage function, FN has been frequently used in biomaterial surface modification to favor implant integration and cell adhesion (142). However, the impact of FN pre-adsorption in controlling macrophage adhesion and cytokine release is often overruled by the material surface chemistry, thereby suggesting the need to better understand the macrophage response to both the material and the proteins that are immobilized on the material (195).

To date, there are no other studies where bioresorbable polymeric TEHV functionalization was evaluated in large animal models. However, in the cardiovascular field, a multitude of growth factors in combination with ECM proteins have been proposed to favor tissue integration and remodeling, as extensively reviewed elsewhere (194, 196). For example, VEGF was demonstrated to significantly inhibit the formation of calcifications in valve interstitial cells, whereas TGFβ1 stimulated calcific nodules *in vitro* (197). Remarkably, the use of FN coating proved to significantly reduce calcification formation despite TGFβ1 administration (197). These results suggest that, to limit the risk of calcifications, specific ECM proteins and growth factors, like FN and VEGF, can be combined to functionalize scaffolds for TEHV applications. Heparin and IL-4 have also been proposed to functionalize polymer. *In vitro* results showed that this functionalization effectively promoted M2 macrophage polarization and created an anti-inflammatory environment in electrospun scaffolds (112). Similarly, the use of a cytokine cocktail (i.e., IL-10 and prostaglandin-E2) was shown to promote tissue integration and to polarize macrophages into M2 pro-healing phenotype, thereby decreasing adverse immune reactions (198), suggesting a potential use of an IL-4 and IL-10 combination to improve material integration and performance.

The use of peptides to functionalize scaffolds for cardiovascular applications has been extensively reviewed elsewhere (149, 199, 200). Peptides such as RGD and REDV are mostly used to favor endothelialization of the construct, as the sequences can be specifically recognized by the endothelial cells (199, 200). However, as discussed in section “Macrophage/extracellular matrix-derived peptide interactions,” their impact on macrophage adhesion and polarization should be further investigated. A stromal cell derived factor 1α (SDF1α)-derived peptide has been proposed as a potential chemokine to attract monocytes and progenitor cells and to modulate tissue remodeling. *In vitro* studies using SDF1α-derived peptides for polymer functionalization showed reduced expression of inflammatory factors, indicating a reduction in inflammatory signaling. *In vivo* implantation of these scaffolds as rat abdominal aorta interposition grafts showed increased presence of macrophages after 7 days, thereby suggesting the potential role SDF1α-derived peptides may have in modulating the immune response (201). However, scaffold functionalization still comes with some limitations, such as the need to ensure the functionality of the bioactive molecule included and the reduced shelf-life of the product due to diffusion of the included protein (55).

## Conclusion

ECM proteins are a potent tool for the immunoregulatory function upon implantation of a tissue engineered heart valve. A multitude of studies suggest the potential role of ECM and ECM-related proteins in regulating macrophage polarization both *in vitro* and *in vivo*. However, their effect on the adaptive or maladaptive remodeling of TEM-based TEHVs should be further considered. Based on these observations, several functionalization approaches for bioresorbable polymeric TEHVs, either using ECM proteins, cytokine/growth factor cocktails, cells capable of secreting ECM-related cytokines, or ECM-derived peptides, have been evaluated both *in vitro* and *in vivo*. However, to this date, none of these approaches have reached clinical translation.

The pre-clinical use of TEM-based TEHVs for pulmonary applications has shown great potential, and their translation to aortic application is awaited. A full understanding of how ECM-based biomaterials specifically engage with the host tissue has not yet been fully elucidated. However, it is crucial to determine how the host responds to the implanted TEM-based biomaterial based on its protein composition, ECM architecture, and 3D structure, to ensure safe clinical translation. In particular, guidelines on what potential contaminants should be completely eliminated or a threshold for potential immunogenic proteins should be outlined and standardized. In addition, extensive ECM characterization of the final product, including identification of pro-inflammatory ECM components and DAMPs, should be performed. With consistent material production and comprehensive regulations on decellularized biomaterials, TEM-based TEHVs may soon become the next-generation heart valve prosthesis.

## Author contributions

NP, MM, and EF drafted the manuscript. All authors reviewed the text and provided critical input to the manuscript.

## Abbreviations

αSMA: α-smooth muscle actin
CAVD: calcific aortic valve disease
COL: collagen
DAMP: damage-associated molecular pattern
ECM: extracellular matrix
FGF: fibroblast growth factor
FN: fibronectin
IL: interleukin
MMP: matrix metalloproteinases
P4HB: poly-4-hydroxybutyrate
PDGF: platelet derived growth factor
PGA: polyglycolic acid
RTM: resident tissue macrophage
TEHV: tissue engineered heart valves
TEM: tissue engineered matrix
TGFβ1: transforming growth factor-β1
VEGF: vascular endothelial growth factor.
